# Abiotic Stress in Rice: Visiting the Physiological Response and Its Tolerance Mechanisms

**DOI:** 10.3390/plants12233948

**Published:** 2023-11-23

**Authors:** Bhaskar Sarma, Hamdy Kashtoh, Tensangmu Lama Tamang, Pranaba Nanda Bhattacharyya, Yugal Kishore Mohanta, Kwang-Hyun Baek

**Affiliations:** 1 Department of Botany, Dhemaji College, Dhemaji 787057, Assam, India; bhaskarsarma252@gmail.com; 2 Department of Biotechnology, Yeungnam University, Gyeongsan 38541, Gyeongbuk, Republic of Korea; hamdy_kashtoh@ynu.ac.kr (H.K.); orgchemtensa@ynu.ac.kr (T.L.T.); 3 Department of Botany, Nanda Nath Saikia College, Titabar 785630, Assam, India; pranabananda_01@rediffmail.com; 4 Nano-Biotechnology and Translational Knowledge Laboratory, Department of Applied Biology, School of Biological Sciences, University of Science and Technology Meghalaya, Techno City, 9th Mile, Ri-Bhoi, Baridua 793101, Meghalaya, India; 5 Centre for Herbal Pharmacology and Environmental Sustainability, Chettinad Hospital and Research Institute, Chettinad Academy of Research and Education, Kelambakkam 603103, Tamil Nadu, India

**Keywords:** abiotic stress, drought, physiology, rice, tolerance

## Abstract

Rice (*Oryza sativa* L.) is one of the most significant staple foods worldwide. Carbohydrates, proteins, vitamins, and minerals are just a few of the many nutrients found in domesticated rice. Ensuring high and constant rice production is vital to facilitating human food supplies, as over three billion people around the globe rely on rice as their primary source of dietary intake. However, the world’s rice production and grain quality have drastically declined in recent years due to the challenges posed by global climate change and abiotic stress-related aspects, especially drought, heat, cold, salt, submergence, and heavy metal toxicity. Rice’s reduced photosynthetic efficiency results from insufficient stomatal conductance and natural damage to thylakoids and chloroplasts brought on by abiotic stressor-induced chlorosis and leaf wilting. Abiotic stress in rice farming can also cause complications with redox homeostasis, membrane peroxidation, lower seed germination, a drop in fresh and dry weight, necrosis, and tissue damage. Frequent stomatal movements, leaf rolling, generation of reactive oxygen radicals (RORs), antioxidant enzymes, induction of stress-responsive enzymes and protein-repair mechanisms, production of osmolytes, development of ion transporters, detoxifications, etc., are recorded as potent morphological, biochemical and physiological responses of rice plants under adverse abiotic stress. To develop cultivars that can withstand multiple abiotic challenges, it is necessary to understand the molecular and physiological mechanisms that contribute to the deterioration of rice quality under multiple abiotic stresses. The present review highlights the strategic defense mechanisms rice plants adopt to combat abiotic stressors that substantially affect the fundamental morphological, biochemical, and physiological mechanisms.

## 1. Introduction 

Rice (*Oryza sativa* L.), a species of Poaceae, is a ubiquitous staple food worldwide, offering vital nutrients, including carbohydrates, thiamin, folate, calcium, iron, pantothenic acid, and energy [1,2]. Due to the global significance of this economically essential crop in supporting growing human populations and meeting extensive nutritional needs, improving grain production and quality standards is becoming increasingly important [3,4]. Although yields have plateaued in the cultivation of most cereals, including rice, in recent decades, climate change is a significant challenge that greatly influences breeders’ decisions regarding productivity and quality issues [5]. In the coming decades, persistent negative impacts of climatic change and global warming can cause shifts in the severity, duration, and frequency of abiotic stress in rice farming, jeopardizing agricultural sustainability and global food security [6]. By 2050, it is anticipated that global warming and changes in the climate will lower irrigated rice production by 7%, while the yields of rainfed rice will likely decline by 6% and, more conservatively, up to 2.5%, respectively [7]. Various strategies have been adopted in climate-resilient agriculture to promote long-term sustainability. The Green Revolution brought a substantial increment in rice productivity across the globe through the usage of promising and high-yielding rice varieties and the implementation of modern farming techniques like drip irrigation, biofertilizers, biopesticides, and usage of recommended doses of plant protection formulations (PPFs) [8].

**Figure 1:**
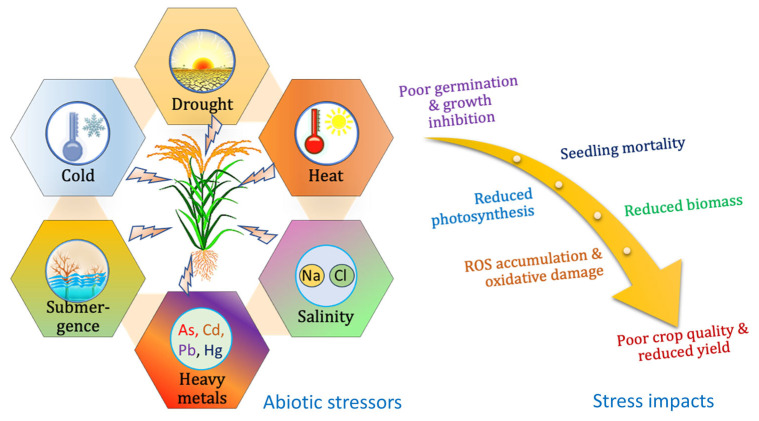
Effects of different abiotic stresses on rice.

Rice farming is under continuous exposure to a broad category of biotic (pathogen invasion and insect infestations) and abiotic (extreme temperatures, drought, cold, heavy metal toxicity, and salinity) stress-related factors leading to serious agricultural issues like poor grain production and quality deterioration [9]. Figure 1 depicts different abiotic stress-related factors that negatively impact rice farming considerably.

Heat stress and drought are major abiotic stressors that interfere with rice’s physiological, molecular, biochemical, and morphological responses, resulting in massive crop losses and compromises in quality [10]. It has become apparent that frequent exposure to high temperatures during rice cultivation appears to have detrimental effects in various tropical and subtropical countries, including India, China, Bangladesh, Pakistan, Thailand, and several African countries. This includes substantial declines in yield and quality, which can be attributed to the sudden occurrence of pollen sterility and loss of fertility [11]. According to Oladosu et al., frequent exposure to drought is detrimental to brown and milled rice, as it can drastically reduce the quality of grain production to a great extent [12]. On the other hand, a rise in temperature leads to a rise in humidity, making spikelets sterile [13]. The flower buds cannot mobilize essential nutrients like carbohydrates and derived products when subjected to extreme heat stress.

Chilling stress is another influential environmental stress that significantly impacts the rice plants’ normal growth and development, including the percentage of seeds that successfully germinate, the vigor of seedlings, the formation of tillers, the reproductive capacity of plants, and the maturity of grains [14]. Similarly, under salinity stress, invasive apoplastic ion transport drives Na+ uptake into rice shoots [15]. Likewise, the submersion of plants can have detrimental effects on various physiological processes, including oxygen and carbon dioxide exchange, light availability, and nutrient absorption. These adverse conditions can hinder the process of photosynthesis, exhaust energy reserves, and eventually lead to growth impairment or the mortality of plants [16]. According to Suwanmontri et al., rice farming under rainfed lowland ecosystems is severely affected by intense and rapid exposure to abiotic stressors, leading to significant damage both in terms of quality and quantity [17]. Furthermore, plants exposed to high amounts of heavy metals experience a decrease or complete halt in metabolic activities and exhibit morphological abnormalities, ultimately leading to a reduction in crop yield [18].

To adapt to these abrupt changes in environments, plants have established intricate response mechanisms for detecting environmental signals and displaying appropriate physiological, morphological, and biochemical adaptations. Abiotic stressors can trigger the up- or downregulation of various genes, activating or inhibiting multiple signaling pathways and enhancing the plant’s tolerance to different environmental challenges [19]. Therefore, a complex interaction of signaling cascades is required at the molecular level to recognize external stimuli and the subsequent awakening of defense mechanisms [16]. In recent years, significant advancements have been made in our understanding of how plants respond to abiotic stresses. This progress can be attributed to contributions made in plant physiology, genetics, biotechnology, and molecular biology. By building upon the existing knowledge of stress tolerance mechanisms in rice cultivars, it is possible to develop novel gene pools that exhibit enhanced resistance to abiotic stresses [20]. In light of the preceding, this review aims to assess the biochemical, physiological, and morphological responses of rice to different abiotic stimuli and identify the process parameters used to generate rice varieties that are tolerant to abiotic stress.

## 2. Morphophysiological and Biochemical Impacts and Tolerance Mechanisms in Response to Different Abiotic Stressors

### 2.1. Drought Stress

The environment has witnessed several persistent repercussions from global climate change, like alterations to the growing season, patterns of rainfall, severe droughts, and soaring temperatures. A significant impact of these changes is the serious threat posed to global rice production by drought stress [21]. Statistics show that 42 million hectares of rice in Asia are occasionally or frequently vulnerable to drought, significantly reducing yield [22,23,24]. According to Lafitte et al., rice suffers economic losses of 48–94% during the reproductive stage due to water stress and another 60% during the grain-filling stage [25]. Reduced cell development, elongation, expansion, and the disruption of plant antioxidant activity triggered by the buildup of reactive oxygen species (ROS) are all ways that drought stress affects rice yield [26].

#### 2.1.1. Morphophysiological and Biochemical Responses to Drought Stress

Plants have different strategies to deal with drought, which include escape, avoidance, and tolerance. Escape involves adapting to a shorter life cycle or growing seasonally to reproduce before the environment becomes dry [27]. Avoidance focuses on maintaining a high water potential in plants by reducing water loss through stomatal control and having a well-developed root system for water uptake [28]. Tolerance, on the other hand, involves limiting the number and size of leaves in response to water scarcity, but this strategy can result in reduced yield [29]. Rice production is particularly impacted by three typical types of droughts: early water stress, which delays the transplantation of seedlings; mild intermittent stress with cumulative impacts; and late stress, which affects late-maturing varieties [30]. The root–canopy ratio, plant height, and dry weight decrease upon water scarcity exposure. Especially at the flowering stage of rice, the rate of photosynthesis, stomatal conductance, rate of transpiration, water potential of leaves, and the air–leaf temperature gap all experience a substantial decline [23]. During the reproductive stage, rice is highly susceptible to water stress, significantly reducing grain production with a drastic decrease in the number of whole grains and spikelets per panicle [31]. The major plant part that detects changes in soil conditions are the roots, which also play a pivotal role in how plants react to water stress. When studying rice root systems under drought stress, a significant positive association was observed between root diameter, depth, and overall plant health and vitality. In drought, plants lengthen their roots to use the water in the soil more efficiently [32]. In response to the drought, rice’s root length increases, enabling the plants to access deeper water reserves in the soil. Additionally, there is a notable reduction in the diameter of nodal roots, leading to the development of relatively finer roots that aid in resource conservation [33]. Many upland *japonica* rice cultivars can withstand drought because of their vast and deep root systems. In contrast, the indica subspecies of rice often experience a reduction in their growth period [34]. Rice is less adapted to water-scarce circumstances than other cereal crops. Upland rice cultivars’ deep root systems are considered good at sustaining yields under drought conditions. In contrast, lowland rain-fed rice crops are susceptible to fluctuating soil water levels, and specific genotypes have adapted to these circumstances by promoting root growth even before and throughout drought [35]. According to Banoc et al., rice plants with well-established root systems exhibit greater water stress resilience and can maintain productivity even under such conditions [36]. Root growth takes precedence over shoot growth when there is a water shortage. Notably, there is a significant disparity in the rate of sap leakage from the root network between rice genotypes that are tolerant to drought and those that are susceptible to it [37].

The rolling of leaves is an adaptive mechanism against water deficiency. This adaptation benefits plants in times of water scarcity and low soil moisture, as it effectively reduces transpiration rates and helps maintain a favorable water balance within plant tissues [38]. As the intensity of the drought stress increases, rice leaves often exhibit varying degrees of leaf rolling. Broader-leafed *indica* rice cultivars perform better in drought conditions than shorter, narrower-leafed varieties regarding biomass, stomatal conductance, and transpiration efficiency [39]. Furthermore, to sustain turgor conditions, plant cells subjected to drought attempt to regulate their osmotic potential by accumulating specific osmolytes. One of the most well-known osmolytes, proline, functions as a mediator in osmotic control to protect the cell against ROS while maintaining the integrity of the plasma membrane. Accumulation of proline is linked to increased resistance to stress [40].

Photosynthesis, a crucial metabolic process that regulates the growth and yield of crops, is influenced by drought and water stress. When water is scarce, the relative water content in plants is reduced. In response, plants employ water-saving strategies such as closing stomata, which reduces the intake of CO_2_, transpiration rate, and gaseous exchange and impedes electron transport, leading to the accumulation of ROS [41,42,43]. Drought stress limits the efficient operation of photosystems I and II (PSI and PSII), disrupts the function of rubisco, and hinders the electron transport chain and ATP synthesis [26,44]. In drought conditions, the efficiency of photosynthetic pigments such as carotenoids, phycobilin, and chlorophyll is diminished. This leads to insufficient absorption of light, inadequate light harvesting, and ineffective photoprotection, eventually leading to limited photosynthesis and a decrease in the production of photosynthates [45,46]. Moreover, carotenoid also has a role in plant signaling during stress; thereby, a reduction in their content can detrimentally affect signal perception during drought stress [47]. Multiple studies have documented the effects of drought stress on the structural integrity of chloroplasts, chlorophyll production, and photosynthesis. When subjected to drought stress, chloroplasts change shape, transitioning from oval to nearly round. Additionally, they move from the cell wall toward the center of the cell, and the thylakoids within the chloroplasts become disorganized [48]. Another study observed irregularly shaped chloroplasts with swollen thylakoids in response to drought stress [49]. The severity and duration of the stress and the specific plant species or genotype determine the extent to which chloroplast integrity is affected [50]. Drought stress leads to the accumulation of ROS, predominantly in chloroplasts and to some extent in mitochondria, resulting in oxidative stress [51]. Furthermore, ROS produced in the chloroplasts of water-stressed plants can negatively regulate the expression of genes related to photosynthesis and chlorophyll production via retrograde signaling [52,53].

Direct or indirect oxidative stress in water scarcity conditions causes cell membrane lipid peroxidation in plants, which in turn stimulates a cascade of physiological and biochemical changes with the potential to disrupt metabolism and negatively impact crop yield and quality [54]. During drought stress, the plant’s ROS overproduction causes an abnormal decrease in photosynthetic electron chains [55]. Various ROS, such as hydroxyl radical (HO^·^), hydrogen peroxide (H_2_O_2_), and superoxide anion (O_2_^−^), are generated by multiple cell organelles. These ROS trigger oxidative damage to cellular components, DNA fragmentation, the suppression of enzyme activity, and lead to lipid and protein peroxidation. They also initiate programmed cell death pathways, ultimately leading to cell death. Antioxidants are vital plant nutrients that scavenge ROS. Therefore, enhancing the expression of antioxidants boosts the rice plants’ ability to withstand drought. Non-enzymatic antioxidants such as ascorbate (AsA), tocopherol, and glutathione (GSH), are different from catalase (CAT), glutathione reductase (GR), ascorbate peroxidase (APX), superoxide dismutase (SOD), and monodehydroascorbate reductase (MDHAR), which are enzymatic antioxidants. The metabolic processes of SOD, CAT, peroxidase (POD), and soluble sugars were elevated in drought-tolerant rice cultivars, whereas malondialdehyde (MDA) level was reduced [56]. At the time of the filling phase, the drought would swiftly increase the activities of POD and CAT while slightly decreasing SOD activity, reducing AsA and GSH contents, and maintaining low levels of H_2_O_2_ and MDA. It is commonly accepted that drought causes increased POD and CAT activities of leaves [33]. The removal of H_2_O_2_ is significantly aided by the use of ascorbic acid, which is an essential antioxidant. During the ascorbic acid-glutathione cycle, APX employs two of the ascorbic acid molecules to catalyze the breakdown of H_2_O_2_ into water. This reaction was followed by the synthesis of monodehydroascorbate. As rice’s drought stress increases, the AsA content of functional leaves drops [57]. Enhancing the content of naturally occurring antioxidants (both enzymatic and non-enzymatic) could be a tactic to lessen or stop oxidative damage and boost plant resilience to drought. During drought, redox-sensitive flavonoids and phenolic acids are synthesized to counteract ROS and bind transition metal ions required for the Fenton reaction [58]. Redox-sensitive phenolic acids (protocatechuic acid, gentisic acid, syringic acid, gallic acid, caffeic acid, salicylic acid (SA), and p-coumaric acid) and flavonoids (rutin, catechin, kaempferol, quercetin, naringin, apigenin, and myricetin) provide drought-tolerant rice cultivars with the capacity to sustain redox homeostasis [59]. Polyamines, which are small molecules with a positive charge, affect rice’s adaptation to stress from drought. Some polyamines identified in plants include putrescine, spermidine, and spermine [60]. They can interact with several signaling networks and control homeostasis, osmotic potential, and membrane stability. When rice plants are subjected to drought stress, there is an elevation in polyamine levels, which is associated with enhanced photosynthetic activity, decreased water loss, and improved ability to detoxify and adapt to osmotic stress [61]. Carotenoids are crucial members of the antioxidant defense system because they prevent the synthesis of singlet oxygen, stabilize triplet chlorophyll in tissues under stress, and shield plants from oxidative damage. As a result, rice’s carotenoid content rises to counteract oxidative stress [62].

#### 2.1.2. Molecular Response to Drought Stress

**Figure 2:**
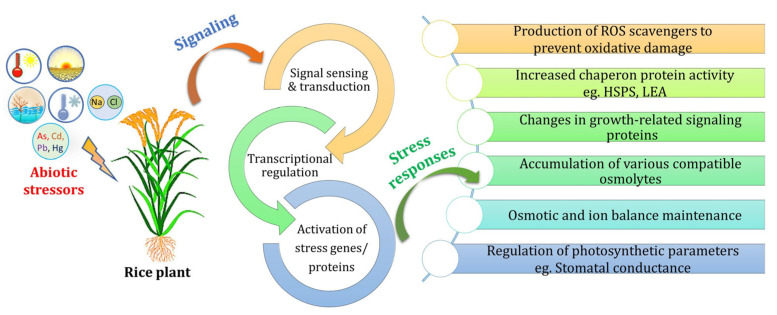
A simplified diagram illustrating how rice plants respond to various abiotic stresses. The overall signaling pathways in plants are triggered when they perceive signals related to abiotic stress, leading to the activation of stress responses.

Rice plants have developed complex mechanisms to survive different abiotic stresses. These mechanisms allow them to adapt or avoid stress by responding optimally. Abiotic stressors are often interconnected and cause damage to plant cells, resulting in oxidative stress [63]. When plants encounter stress, membrane receptors detect the initial signals and transmit them to initiate transcription. This process is controlled by hormones, transcription factors (TFs), and transcription factor-binding proteins (TFBPs). These factors work together to activate stress-responsive mechanisms, repair damaged proteins and membranes, and restore homeostasis [64] (Figure 2). Inadequate response at any stage of the signaling and gene activation process can lead to permanent alterations in cellular equilibrium, breakdown of functional and structural proteins and membranes, and ultimately, cell death [65].

To combat water scarcity, drought stress in rice activates both abscisic acid (ABA)-dependent and ABA-independent signaling pathways [66]. It works via extensive and intricate signaling pathways to regulate drought stress. This involves adjusting the physiological, biochemical, and molecular attributes of the rice to improve the root’s ability to acquire more water and the stomata’s ability to lose less water. This adaptation helps the plants cope with water scarcity stress. Plants respond to drought stress by narrowing their stomata to reduce water loss, improve water utilization efficiency, and enhance their chances of survival [67,68]. ABA governs the movement of stomata to lessen the transpiration rate under drought stress [69,70,71]. ABA receptor, OsPYL/RCAR5, has been demonstrated to exert a positive regulatory effect on the expression of genes that are responsive to abiotic stress, and overexpressing the *OsPYL/RCAR5* gene additionally enhanced transgenic rice’s ability to withstand drought [72]. Research has demonstrated that rice *DREB* transcription factors are essential controllers of ABA-independent drought responses. Rice cultivars that overexpress *OsDREB1F* exhibited improved drought tolerance, indicating that this gene mediates the ABA-dependent pathway [73]. When rice undergoes drought stress, the root system improves cuticle resilience and boosts the number, density, and depth of root hairs [74]. One key component in achieving that is *DRO1*, a combined quantitative trait locus (QTL) linked to root depth, which is upregulated in response to drought stress, promotes deeper growth of roots, and enhances tolerance against drought [75]. It also regulates the elongation of cells of the root tip, asymmetric growth, and bending of the root tip. When transformed with *DRO1*, rice cultivars with shallow roots become drought tolerant by establishing a deeper root system [75]. Drought resistance also depends on genes related to osmotic adjustment, equilibrium of stomatal activity, water-use effectiveness, phytohormones, and root and shoot biomass. Various genes, like *OsPYL/RCAR5* and *EcNAC67*, cause delayed leaf rolling and increased root and shoot mass under drought stress [72,76]. Drought resistance in rice is improved by *EcNAC67* overexpression. When exposed to water stress, in comparison to non-transgenic *ASD16*, transgenic plants displayed delayed leaf rolling signs. Additionally, they revived quickly after re-watering, retained a 20% higher relative water content in the leaves, and experienced a less pronounced decline in plant height and yield [76]. Research studies revealed that the *DSM1* gene, a Raf-like MAPKKK, might modulate ROS scavenging to mediate drought responses in rice [77]. Table 1 presents a summary of key genes associated with drought resistance in rice.

It has been noted that seed priming is an effective strategy to reinforce the antioxidative defense system and enhance plant stress responses. One study observed a notable increase in antioxidant activity, total phenolic content, and expression of *RD1* and *RD2*, rice drought-responsive genes belonging to the AP2/ERF family in two different rice genotypes, Nagina-22 (known for its drought tolerance), and Pusa Sugandh-5 (known for its drought sensitivity). This upregulation was observed when the seeds of these genotypes were primed with different plant hormonal or chemical elicitors, such as methyl jasmonate, SA, and paclobutrazol, under drought stress [98]. Rice that has been colonized by *Trichoderma harzianum* isolates is drought tolerant, grows faster, and experiences a delay in the effects of drought [99]. Colonization boosts rice’s ability to acquire and store water and root growth. In colonized plants, there is a lesser increase in the concentration of stress-induced metabolites.

### 2.2. Heat Stress

Global food security is now seriously threatened by heat stress brought on by a fast-changing climate. When the temperature rises above a specific point and continues for a while, it is said to be under heat stress, which can permanently harm plant growth and development [100]. Without effective adaptability, CO_2_ fertilization, and genetic development, it is predicted that every one-degree rise in the global mean temperature will result in lower worldwide yields of wheat, rice, maize, and soybeans [101]. Rice can grow normally at temperatures between 27 and 32 °C. Above 32 °C, all phases of growth and development of plants are negatively affected. The flowering stage, however, required a temperature of 33 °C. Heat damage occurs when rice is exposed to air temperatures above 35 °C [102].

#### 2.2.1. Morphophysiological and Biochemical Responses to Heat Stress

Rice has three types of heat stress resistance: defense, avoidance, and tolerance. Heat defense is the mechanism of controlling morphological development and transpiration of leaves to lower the temperature of the panicles and avoid deterioration from scorching temperatures [103]. Heat avoidance includes adjusting spikelet flowering time by shortening the flowering period and early blooming, which is a desirable characteristic for developing heat-resistant rice cultivars [104]. Heat tolerance is the ability to continue generally living in hot temperatures. In response to heat stress, rice adjusts its physiochemical processes, which comprises growth retardation, leaf rolling, the senescence of leaves, and changes to fundamental physiological functions such as photosynthesis, respiration, the permeability of membranes, and ROS, that minimize the pollen sterility [105].

In addition to the hormone synthesis that influences the growth and development of shoots, the roots play essential roles in water intake and nutrients [106]. Although root systems are crucial in helping plants adapt to high temperatures, their thermotolerance mechanism has been less explored. Most of the research has focused on studying the aerial parts of plants [107,108]. Root growth is more susceptible to high temperatures than shoot growth, due to its lower optimal temperature [109]. Typically, when soil temperatures are elevated, a decrease in root growth and physiological activity occurs before the cessation of shoot growth [110]. A study showed that the rice plant roots failed to elongate and divide at a temperature of 43 °C [111]. Heat stress can affect rice plants during most of their vegetative growth stages. When temperatures are consistently high, the potential for seed germination decreases, resulting in a lower germination rate and weaker seedling growth [112]. When exposed to heat stress (42–45 °C), the seedlings experience increased water loss, wilting and yellowing of leaves, hindered growth of roots and seedlings, and in severe cases, death of the seedlings [102,113]. Similarly, rice seeds failed to germinate upon continuous exposure to a constant temperature of 43 °C [114]. In addition, another study found that rice plants died in the initial vegetative phase when exposed to a constant air temperature of 40 °C and high levels of CO_2_ (700 ppm) [115]. Furthermore, when a sequence of distinct heat stress treatments was applied to rice seeds, young seeds, in particular, were the most vulnerable in the initial two days following flowering [116].

Rice plants are more vulnerable to heat stress during the reproductive stage than the vegetative stage, including initiation of panicle, development of male and female gametophytes, anthesis, pollination, and fertilization [117,118]. Under heat stress (40 °C day/35 °C night) for 15 days, rice output per plant was 86% lower overall, and the panicle number was roughly 35% lower [119]. In *japonica* rice, compared to *indica* rice, heat stress significantly impacts the number of tillers and panicles [120]. When the rice plant enters the flowering stage, it becomes highly vulnerable to elevated temperatures. The second-most vulnerable stage appears around nine days before blossoming. Significant rises in temperatures during anthesis cause a high proportion of spikelets to be sterile. During the grain-filling stage, heat stress has been observed to impact the quality of rice negatively. This is evident through a decrease in palatability, an unfavorable grain appearance, and an increase in grain chalkiness [121,122,123,124]. The presence of chalky kernels is considered the most prominent indication of heat stress during this particular phase of rice development. During the panicle-initiation stage, heat-stressed plants experience a decrease in non-structural carbohydrates, underdeveloped vascular bundles, and smaller glumes, ultimately reducing grain weight [125]. The total grains and rice production percentage declines as nighttime temperatures rise. White immature kernels are formed when rice plants endure exposure to high temperatures at the ripening stage, disrupting the carbohydrate sink–source balance. The increased rhizosphere temperature causes the total dry weight of super rice to decrease by 16.26% [126].

A reduction in the stomatal aperture size, the xylem in the leaves, and an increase in the trichome density on both surfaces are additional examples of common adaptive responses to heat stress [127]. Photosynthesis is a crucial biochemical function in plants that is most susceptible to heat. The main sites of injury at high temperatures in chloroplast are light-dependent reactions in the thylakoid membrane and carbon fixation reactions in the stroma [128]. High temperature has a strong affinity for the thylakoid membrane. Significant changes in chloroplasts include changed thylakoid structural arrangement, loss of grana stacking, and grana swelling during heat stress. Heat shock decreases the number of photosynthetic pigments. At extreme temperatures, the enzymatic activities of invertase, ADP-glucose pyrophosphorylase, and sucrose phosphate synthase are diminished, leading to a substantial decrease or complete cessation of the function of PSII [129,130].

Heat stress-induced imbalance in metabolic activities, including photosynthesis and respiration, results in a rise in ROS or a fall in the cell’s efficiency to scavenge oxygen radicals. When exposed to high temperatures, rice anthers produce much more ROS, decreasing floret fertility and pollen viability [131]. MDA, a reliable indication of free radical damage to cell membranes, is produced when membrane lipids under heat stress undergo peroxidation. Increased lipid peroxidation demonstrated that oxidative stress frequently developed in rice leaves following exposure to high temperatures [132]. Various enzymes and metabolites take part in the antioxidant defense framework. The antioxidant enzymes, such as SOD, APX, CAT, GR, glutathione peroxidase (GPX), and peroxiredoxins, assist in shielding the cells from an accumulation of ROS. Furthermore, Phenolic chemicals can remove ROS, neutralize singlet and triplet oxygen, or break down peroxides. Moreover, the GSH molecule has a crucial function in protecting the photosynthetic system [133].

#### 2.2.2. Molecular Response to Heat Stress

Heat stress signals are sensed through numerous heat shock transcription factors (HSTFs) and proteins. Various genes related to Ca^2+^ homeostasis, ROS, lipid metabolism, and phytohormones are activated to trigger the response against heat stress [134]. In rice, a large number of high-temperature-related genes, including stress-related transcription factors (TFs), HSTFs, and heat shock proteins (HSPs), have been cloned. These genes are involved in heat stress-related temperature sensing and response [135] (Table 2). *OsHSP26.7*, for instance, encodes an HSP that shields chloroplasts from oxidative damage brought on by extreme heat and ultraviolet radiation [136]. Similarly, under the *HSP101* promoter, *OsWRKY11* encodes a TF with a WRKY domain that can dramatically increase rice’s tolerance towards heat and drought [137]. Furthermore, a NAC TF called *SNAC3* mediates ROS metabolism, and OsMYB55 TF in rice significantly improves tolerance to high-temperature and increases grain yield [138,139]. The *HYR* gene is a crucial regulator that can directly activate photosynthesis and can control downstream genes involved in carbon metabolism as well as morphology and physiology during drought and heat stress, maintaining the yield of rice [140]. The cytoskeleton plays a vital role in the ability of organisms to tolerate and adapt to stressful conditions. In the case of rice, a specific intermediate filament called *OsIF* has been identified as being particularly important in mitigating the implications of heat and salinity stress on the photosynthetic apparatus and overall crop yield [119]. Additionally, several enzymes, including glutamate decarboxylase and glutamine synthase, are some of the additional key factors that produce stress-related amino acids that aid rice in tolerating extreme heat [139,141]. A mitochondrial lipase known as EG1 can activate the expression of floral organ genes during high temperatures, thereby preserving the consistency of floral organ growth [142]. Table 2 presents a summary of key genes associated with heat stress tolerance in rice.

As a key defense against heat stress, plants accumulate soluble carbohydrates like glucose and fructose as well as non-soluble sugars like starch [156]. Under acute heat stress, the expression of OsSUT1, a sucrose transporter, is elevated, which results in increased sugar buildup and reduced photosynthesis [157]. Tolerance to high temperatures in plants is greatly influenced by the accumulation of certain metabolites. Under intense heat, the MYB55 TF in rice controls the expression of downstream glutamate dehydrogenases GAD3 and glutamine synthase OsGS1.2, thus promoting the buildup of stress-related amino acids like gamma-aminobutyric acid (GABA) and L-glutamic acid [139]. The analysis of the temporal transcriptome of germinating seeds subjected to heat stress at 35 °C reveals that the early response to heat stress is mediated by the Inositol-requiring enzyme 1 (IRE1)-mediated endoplasmic reticulum (ER) stress response and the jasmonic acid (JA) pathways. As JA promotes the spliced form of *OsbZIP50*, a gene marker linked to the IRE1-specific pathway, it is hypothesized that the rise in JA concentration levels during heat stress may happen before the ER stress response [116]. Numerous genes associated with high-temperature responses have been documented, leading to a better understanding of the signaling pathways in which they participate. Nevertheless, the precise molecular processes and regulatory systems underlying sensing of high-temperature signaling and transmission to downstream components remain inadequately recognized, thus necessitating further investigation as the critical area of prospective studies.

Ethylene, a crucial plant hormone, significantly regulates biotic or abiotic stress signaling. In the case of heat stress in rice seedlings, ethylene-mediated signaling has been found to mitigate oxidative damage, preserve chlorophyll levels, and enhance thermotolerance [158]. Specifically, under heat stress conditions, ethylene-mediated signaling controls the mRNA transcripts of certain heat stress transcription factors (HSFs) and genes related to ethylene signaling [125]. Phytohormones are also crucial in controlling how rice yield qualities react to heat stress. Specifically, cytokinin and abscisic acid (ABA) regulate the number of spikelets per panicle under high-temperature conditions. Additionally, gibberellin and indole-3-acetic acid may be associated with spikelet fertility, while indole-3-acetic acid, ABA, gibberellin, and cytokinin regulate grain weight [100].

When exposed to heat stress, foliar sprays of boric acid (25, 50, or 100 mg L^−1^) or sodium borate (50 mg L^−1^) substantially boosted net photosynthetic rates in comparison to untreated plants [159]. The use of foliar borate compounds on seedlings experiencing heat stress led to a decrease in oxidative damage, as indicated by the reduction in the levels of leaf MDA and proline synthesis and an enhancement in the photochemical efficiency of PSII.

### 2.3. Cold or Low-Temperature Stress

Rice is sensitive to cold, especially during the germination process, which causes significant economic losses. The dynamics of the crop’s growth are negatively impacted by cold stress in temperate and high-altitude rice-growing regions in the tropics and subtropics [160]. Cold stress has detrimental consequences on rice, such as decreased seedling growth, poor germination, constrained leaf expansion, chlorosis, and wilting. Necrosis, or tissue death, is the final impact of these factors [161].

#### 2.3.1. Morphophysiological and Biochemical Responses to Cold or Low-Temperature Stress

In circumstances of cold stress, the growth of rice shoots and roots is hindered in terms of length, fresh and dry weight, and protein content [162]. A research study found that when exposed to cold stress, the root growth and developmental characteristics of various genotypes of rice decreased, ranging from 2% to 87% [163]. Furthermore, when rice is subjected to cold stress during the vegetative stage, the leaves begin to yellow, the plant grows shorter, and the number of tillers decreases [164]. Rice’s ability to germinate, as well as its coleoptile and radicle growth, is significantly reduced by low temperatures. Inhibition of seed germination and growth retardation or death of the seedlings cause a decline in crop yield [165]. The reproductive phase of rice, specifically in the post-meiotic stages of anthers, has a pronounced impact on pollen production due to cold stress [166]. In addition, cold temperatures during the immature microspore stage of rice anthers lead to heightened protein degradation. Other effects of cold stress comprise damage to the photosynthetic apparatus, including modifications to the number of chloroplasts, ultrastructure, light-harvesting chlorophyll antenna complexes, modified grana arrangement, and lamellar structures [164,167]. Thus, there is a shortage of plant energy resources since cold temperatures generally slow photosynthetic processes. This is due to the reduced activity of several enzymes involved in tetrapyrrole metabolism and the down-regulation of gene expression, which affects chlorophyll production [164]. The circadian clock is crucial for rice’s reaction to chilling stress. Night chilling stress affects leaf chlorophyll metabolism and PSII more severely than its daytime equivalent [168]. Additionally, nitrogen intake has often been found to be restricted by chilling stress in rice [169]. Numerous studies have shown that stress caused by low water temperature reduces nitrogen absorption [170,171]. This could be attributed to the decreased activity of enzymes and transporters in the roots under such conditions.

Plants have developed advanced mechanisms to prevent damage caused by cold temperatures. One such mechanism is cold acclimation, where plants exposed to mild cold temperatures for a short period become more resistant to following freezing stress [172,173]. During cold acclimation, various physiological, biochemical, and molecular transformations take place. These include the activation of antioxidant systems, the production and buildup of cryoprotectants, and the implementation of mechanisms that safeguard and stabilize cell membranes [174]. To keep the cell membrane stable, the content of unsaturated phospholipids in the membrane increases. Additionally, cells store osmotic molecules rich in sucrose and proline, as well as antifreeze proteins, which help to retain water molecules [175]. Plants synthesize various proteins such as late embryogenesis abundant (LEA), anti-freezing proteins (AFP), and cold shock proteins (CSP) to increase their tolerance to cold stress [176,177]. Lower molecular-weight solutes, soluble sugars, and proline act as osmoprotectants to shield plants from cold-induced damage. Similarly, the accumulation of protective proteins like LEA, AFPs, and CSPs during cold acclimation is crucial for enhancing cold tolerance in plants [178]. The acclimation mechanism is crucial for improving the ability of plants to withstand cold temperatures. Even plants that are sensitive to cold, like rice, can adapt to chilling conditions [179,180]. Freezing-resistant plants also adapt through cold acclimation, where they are exposed to temperatures slightly above freezing. Under these conditions, aquaporins play a key role in regulating the water uptake mechanism and the permeability of cell membranes [181,182,183,184]. Various studies have shown that aquaporins are functionally important in controlling the hydraulic conductivity of roots (Lpr) [180,185,186]. It has also been demonstrated that the decrease in water uptake in rice under cold stress is associated with a decrease in aquaporin expression [187].

Furthermore, the presence of low temperatures can result in the buildup of ROS and H_2_O_2_. This accumulation can subsequently lead to leakage of electrolytes, lipid peroxidation, and damage to the cell membrane [188]. This can be observed through the rise in levels of MDA. The breakdown of polyunsaturated lipids to MDA is one possible way ROS can damage cells and tissues [188,189]. Plants contain a variety of antioxidant systems to prevent catastrophic breakdown of protein and lipid components when under stress. Antioxidants like CAT, POD, 2,2-diphenyl-1-picrylhydrazyl, and SOD can compete against ROS generation in rice under cold stress due to their high stability and pace of rising [164]. A study on rice cultivars under cold stress found that cultivars with a faster growth rate had greater H_2_O_2_ levels in the shoots but lower levels in the roots. However, this was reversed in the case of rice cultivars with a low growth rate. Moreover, the roots had higher MDA concentrations and electrolyte leakage due to cell damage than the shoots under cold stress. Cold stress boosts SOD and CAT activities in the rice roots [162]. These biochemical characteristics can be used as a selection marker for breeding and adjusting rice crops with enhanced cold tolerance.

Glutamic acid (Glu) is essential in the amino acid metabolism of plants and is involved in vital metabolic processes during abiotic stress [190]. These functions include the production of proline and gamma-aminobutyric acid (GABA), which are essential for plants’ defense systems [191]. Under cold stress, GABA, proline, and soluble carbohydrates like glucose and sucrose buildup in rice and work as osmoprotectants to prevent damage from dehydration and freezing [192,193]. The findings suggest that GABA and proline could improve plants’ ability to withstand cold temperatures.

#### 2.3.2. Molecular Response to Cold or Low-Temperature Stress

Rice plants must maintain the stability of their cell membranes, their levels of chlorophyll and fluorescence, the initiation of ROS defense mechanisms, and the accumulation of osmolytes to withstand cold stress [194]. During cold stress, COLD1 and CIPK sense cold-related stress signals, and several genes relating to osmoprotectants and phytohormones are modulated. To facilitate cold sensing and extracellular Ca^2+^ influx at low temperatures, COLD1 has been demonstrated to interact with the rice G protein α subunit 1 (RGA1) [195]. Rice CBL-interacting protein kinase 7 (OsCIPK7), in addition to *COLD1*, is believed to recognize cold stress cues by controlling the configuration of its kinase domain and the influx of Ca^2+^ [196].

At low temperatures, endogenous ABA levels rise, and expression of ABA-responsive genes is activated, strengthening plant tolerance to cold stress. Overexpression of the *OsPYL9* (an ABA receptor), which positively modulates ABA signaling, can dramatically increase rice’s ability to withstand low temperatures [197]. In addition to the fundamental component PYL-PP2C-SnRK2-ABF, the ABA signaling pathway also involves nitric oxide (NO), ROS, Ca^2+^, phospholipid molecules, and other kinases, like MAPK [198]. The mitogen-activated protein kinase OsMAPK3 elevates trehalose content and strengthens rice adaptation against cold stress [199]. Table 3 presents a summary of key genes associated with cold stress tolerance. Although there has been a significant advancement in cold stress tolerance, little is known about single-cell responses in rice plants.

Abiotic stressors can be effectively reduced using nanoparticles. Zinc oxide nanoparticles (ZnO NPs) applied topically considerably reduce the chilling stress experienced by rice seedlings, resulting in increased plant height and root length and enhanced dry biomass. With the decreased concentration of H_2_O_2_ and MDA, in addition to higher activities of the key antioxidative enzymes like SOD, CAT, and POD, ZnO NPs further restore chlorophyll accumulation and markedly mitigate chilling-induced oxidative stress [221]. Plant melatonin, an organic molecule, has also been demonstrated to be crucial for plant stress adaptation. Melatonin pretreatments boost the non-enzymatic antioxidant content and upregulate the antioxidant enzyme activity in rice. The application of exogenous melatonin reduces rice seedling development inhibition, formation of ROS, MDA, inhibitions of photosynthesis and PSII activities, and cell death brought on by cold stress in rice [222]. Similarly, Teixeira et al. found that rice seed priming with carrot extract greatly speeds up germination and raises the final germination percentage while reducing the damage caused by cold [223].

### 2.4. Submergence Stress

Submergence is a major concern for rice cultivation in lowlands subjected to rainfall and flood-prone regions globally. It is expected to become more common as climate change increases flood threats, particularly in regions impacted by monsoon rains in Asia [224]. Rice plants possess a partially aquatic characteristic, enabling them to thrive in waterlogged or submerged environments for extended periods [225]. Nevertheless, prolonged submersion exposes rice plants to various stresses, such as reduced access to light, decreased gaseous exchange, physical damage, and increased vulnerability to pests. In addition, submergence typically lowers the photosynthesis process, depleting carbohydrate stores and eventually causing the death of the plant [226]. Rice usually comes to be affected by two different types of flooding. The initial type is flash flooding, which arises when the crop is flooded for 1–2 weeks due to a sudden rise in water levels. Another kind of flooding is stagnant flooding, in which the water level rises above 100 cm and stays there for several weeks [227].

#### 2.4.1. Morphophysiological and Biochemical Responses to Submergence Stress

Rice is extremely sensitive to submersion during the germination and early seedling growth stages. When rice seeds are entirely submerged in water, they suffer from hypoxia or anoxia, resulting in poor germination and seedling mortality [228]. The rice plant undergoes numerous morphological and physiological changes as a result of submergence. Rice withstands submersion by growing longer leaf sheaths and blades during the seedling stage and internodes during the vegetative growth stage [229]. Even submergence-tolerant types attempt to expose their leaf tips above the water’s surface if the flooding lasts longer than two to three weeks to ensure their survival [230,231]. When fully submerged, the leaves and stems of the rice plant grow moderately longer to reach the water’s surface. However, there are negative effects from this elongation process that are necessary for post-submergence plant growth [232]. Turbid water reduces the amount of light that may pass through floodwater, which lowers photosynthesis and, as a result, the submerged plant uses its reserve carbohydrate to sustain its metabolism [233]. However, if the depth of flooding is significant and the duration of flooding is prolonged, the plant’s limited ability to perform photosynthesis causes its energy reserves to deplete rapidly, ultimately leading to the plant’s death [234]. The amount of carbohydrates found in plant sections determines a variety’s capacity to withstand submersion [235]. Submergence-tolerant rice cultivators benefit from limited shoot elongation because they preserve carbohydrate reserves, which aid in resuming development after de-submergence. For recovery from submergence shock, carbohydrate availability following flooding is crucial [236]. During periods of flooding, plants are entirely or partially immersed in water. However, when the floodwater recedes, the plants are suddenly exposed to oxygen again. This reoxygenation process can harm plants after being submerged. MDA, O_2−_, and H_2_O_2_ were found to increase in rice plants’ leaves after being submerged for seven days as a sign of oxidative damage [237]. Rice leaves began to dry out when exposed to air oxygen again after being submerged for 7 to 10 days [238]. Due to conserving glucose metabolism during submersion, tolerant rice cultivars on de-submergence exhibit an ascent in fresh biomass. On the other hand, the non-tolerant cultivars’ reserves undergo hydrolysis and are incapable of regeneration. These findings suggest that resistance to several stresses, including submersion, re-oxygenation, and dehydration, is necessary for a plant to survive a flood [239]. Due to frequent oxygen deprivation and low light intensity, submerged plants develop ROS, which, if unchecked, can adversely harm the cellular structure and end in plant death. [240]. The antioxidant defense mechanism is crucial to detoxify ROS and lessen their harmful effects. SOD, APX, and GPX are the substances that are crucial in ROS detoxification [241].

To thrive in submerged environments, rice cultivars employ two growth control techniques: quiescence and escape strategies, both of which rely on ethylene-responsive transcription factors (ERFs). In the quiescence strategy, shoot prolongation is postponed for quite some time (10–14 days) during flash flooding to save carbohydrates [242]. Utilizing conserved carbohydrates, cultivars that can withstand submersion can resume their growth after de-submergence. The escape strategy is adopted by deepwater rice genotypes and involves rapid internode extension to climb above the water level [243]. To implement these strategies, rice has evolved specific anatomical and morphological characteristics. These include the development of adventitious roots, aerenchyma formation, radial oxygen loss (ROL) barrier, and the ability to create a thin film of gas on its leaves. Furthermore, rice plants generate ventilated tissues and ethylene to aid in gas exchange and regulate the programmed death of specific cells in the cortex and epidermis [244,245]. In addition, the growth of adventitious roots regulates the death of epidermal cells utilizing the mechanical energy they produce [246]. When submerged, rice plants rapidly accumulate gibberellic acid (GA), which leads to the elongation of internodes [247]. To protect their roots from oxygen loss, rice plants form an ROL barrier. This barrier extends from the base to the tip of the roots and is located outside the aerenchyma [248]. Various Asian rice cultivars have developed additional characteristics to adapt to prolonged submergence. These traits include aerobic germination and dormancy of leaf elongation during flash floods, and internode elongation during periodic flooding. Certain rice cultivars can withstand being submerged for around 15 days by limiting elongation growth, carbohydrate consumption, and chlorophyll degradation [249,250].

One of the significant regulators of rice’s submergence reactions is ethylene. Owing to physical confinement and active production during stress, this gaseous phytohormone quickly builds up in tissues of submerged plants, inducing various acclimation reactions, such as shoot elongation, development of adventitious root, and glucose metabolism. Deepwater rice encourages internode growth during submersion to project the photosynthetic parts of the plant above the air–water contact [242]. High production rates of ethylene and sensitivity to the hormone mediate this flight response. Lowland rice that can withstand submersion, in contrast, limits the number of carbohydrates it consumes, which encourages underwater elongation and is used for cell division and elongation. Limited ethylene production and sensitivity are the causes of this tolerance [251]. Aerenchyma, which allows for relatively unimpeded movement of O_2_ from well-aerated shoots to buried roots, is another way lowland rice adapts to soil waterlogging [252]. Inducing a barrier to radial O_2_ loss (ROL) that reduces O_2_ loss to the surroundings can further boost longitudinal O_2_ diffusion along the root apex. Under flooded conditions, these characteristics are used by both lowland and upland different rice species [253].

Unlike flood-sensitive rice types, flood-tolerant rice cultivars utilize energy stores more effectively and maintain higher non-structural carbohydrate (NSC) concentrations in stems and leaves. Additionally, they use anaerobic respiration as a different energy-producing method. Submergence-tolerant rice cultivars decrease shoot prolongation to preserve energy for survival and recuperation following de-submergence. Complete submersion-tolerant rice genotypes maintain their chlorophyll and embrace a strategy of modest growth, shown by reduced elongation when submerged. Because of this, plants can save enough glucose reserves to maintain metabolism while submerged and after the floodwaters have receded [250].

#### 2.4.2. Molecular Response to Submergence Stress

Rice plants implement passive approaches for adapting and avoiding recurring floods. *SUB1A* is a crucial modulator of submergence tolerance, which activates transcriptional modulation of other ERF response factors and *SLR1* [250]. In deepwater rice, the ERF OsEIL1 is stabilized by ethylene accumulation. OsEIL1 binds to the SD1 promoter to boost gene expression. SD1 participates in GA synthesis and affects internode elongation [254]. The GA then increases the expression of the Accelerator of Internode Elongation 1 (ACE1), while DEC1, a protein that prevents internode elongation, sees a decrease in expression [255]. In addition, OsEIL1 also activates the expression of other downstream genes as a result of submergence stress by binding to the promoter sites of *SNORKEL1* (SK1) and *SNORKEL2* (SK2) [247,256]. Table 4 presents a summary of key genes associated with submergence stress tolerance in rice.

A study found that SK1 and SK2 respond during flood stress by encoding response factors associated with ethylene signaling [264]. During submergence, ethylene levels in rice rise, and the expression of SK1 and SK2 elevate, ultimately promoting internode elongation via GA [265,266,267,268]. Functional assessment of ERF-type TFs indicated that they play a role in regulating several physiological and morphological responses to submersion. SUBMERGENCE-1 (*Sub1*) and SK are TF genes that belong to the ERF class [247,249]. Three clusters of related genes, *SUB1A*, *SUB1B*, and *SUB1C*, expressing ERF-like TFs, are found in the Sub1 region of submergence-tolerant cultivars, with *SUB1A* being the most investigated. Systematic genetic analyses showed that *SUB1A* introgression with *SUB1B* and *SUB1C* imparts a strong endurance against submergence and does not alter rice grain quality or production [234,249,250,269]. Additionally, *SUB1A* prevents the development of proteins that loosen and expand cell walls in response to flooding stress, preserving high levels of chlorophyll a and b [270]. Furthermore, *SUB1A* also promotes resistance to oxidative stress by controlling genes that encode ROS-detoxifying enzymes [237].

In soil, silicon (Si) is the second most prevalent element. According to Debona et al., silicon significantly increases plant resilience to various biotic and abiotic stressors [271]. Si treatment improves rice root morphological features and chloroplast ultrastructure to counteract the inhibitory effect of submergence stress by boosting Si absorption, accumulation, and plant biomass. Si also lessens oxidase damage by increasing POD and CAT activity and decreasing MDA concentration, which helps rice recover from submersion stress-related damage [272,273].

### 2.5. Salinity Stress

Salinization is becoming an ever-worsening problem resulting from poor agricultural practices and environmental changes. Salinity is characterized by excessive levels of various salts in the soil, including sodium chloride, magnesium sulfates, magnesium bicarbonates, calcium sulfates, and calcium bicarbonates. When it is young, the rice crop is considered a salt-sensitive cereal, and as it matures, salinity limits the yield’s efficiency [274,275]. Salt stress is particularly detrimental to rice during its early vegetative and reproductive phases. Water, along with toxic ions from the soil, enter the vascular section of the root system via two pathways: apoplastic and symplastic. Through the apoplastic pathway, salt stress causes shoots to accumulate more Na^+^, primarily in mature leaves. A Na^+^/K^+^ symporter called the high-affinity potassium transporter (HKT) controls the movement of Na^+^ and K^+^ within plant cell membranes [276,277]. The potassium uptake is hampered by sodium ions overloading the root’s surface. Na^+^ interferes negatively with K^+^ uptake because it shares the same molecular characteristics as K^+^. When plants come under salt stress, a considerable quantity of Na^+^ enters the plant, elevating the intracellular Na^+^ levels. This has detrimental impacts since Na^+^ competes with K^+^ to activate enzymes and synthesize proteins [278].

#### 2.5.1. Morphophysiological and Biochemical Responses to Salinity Stress

Rice plants exhibit various morphological, physiological, or biochemical changes and symptoms when exposed to high salinity. In extreme cases, they may even perish. Direct accumulated salts interfere with metabolic functions and all key morpho-physiological and yield-related traits, comprising photosynthesis, plant height, root length, tiller number, length of panicle, spikelet count per panicle, filling of grains, and plant biomass. As a result, yield is significantly reduced [279,280,281]. In a salt-sensitive plant, exposure to salinity stress results in pericycle shrinkage and physical damage. Salt stress exposure at the early seedling stage raises the mortality rate of rice leaves [282]. The productiveness of the rice crop under salt stress is greatly impacted by panicle sterility [283].

Salinity generally induces two types of stress in plants: osmotic and ionic stress. Osmotic stress arises when the salt concentration around the plant’s roots exceeds the threshold tolerance level. On the other hand, ionic stress develops when there is a large Na+ inflow into the plant, which raises the salt concentration in older leaves to a toxic level. This leads to higher Na^+^ concentrations in the vacuole and cytoplasm, disrupting metabolic processes and causing cell death [284]. In the beginning, osmotic stress caused by soil salinity restricts plant growth, and later, ionic stress follows. A significant amount of salt in the soil contributes to the first phase, characterized by reduced plant water intake and the subsequent induction of several cellular metabolic processes [285]. Enlargement of cells, cell wall protein synthesis, net photosynthesis, photosynthetically active radiation, stomatal conductance, relative water content, transpiration rate, and pigment degradation are all inhibited during the initial phase whereas the accumulation of compatible solutes and ABA increased [286]. According to research by Cha-umi et al., salt stress caused a significant drop in carotenoid and chlorophyll in rice leaves [287]. During the latter phase, the accumulation of ions (Na^+^ and Cl^−^) is linked to changes in the ions ratio of Na^+^/K^+^ and Na^+^/Ca^2+^. The subsequent increase in ions promotes the synthesis of ROS. The extra ROS generation increases cellular oxidative stress, which upsets the equilibrium between generating and eliminating ROS [288].

Like the majority of plants, rice has developed several defense strategies against salinity stress, such as (i) antioxidant generation for ROS detoxification (ii) ion homeostasis and compartmentation, (iii) osmoprotection through osmolyte regulation, and (iv) programmed cell death [289]. Plants have devised an exquisite antioxidant defense mechanism to scavenge and detoxify ROS to shield the cells from oxidative damage. According to studies, the salt-tolerant rice cultivar Pokkali performed better under salinity stress than the Pusa Basmati (salt-sensitive rice cultivar) in terms of ROS scavenging enzymes like CAT and content of antioxidants like AsA and GSH [290]. In rice plants, the basal area of the leaf can scavenge H_2_O_2_ by boosting the activity of CAT and maintaining higher constitutive levels of APX and GPX than those in the apical region under salinity. Under salt, the GR in the basal area might inhibit O_2_ generation. The apical area can, however, scavenge O_2_ by boosting SOD activity, whereas, under salinity, the activity of H_2_O_2_ scavenging enzymes, including APX and CAT reduced [291]. To prevent the rice from oxidative stress brought on by salt, both enzymatic and non-enzymatic ROS scavenging machinery must work together. A transcriptional cascade in rice roots, which is regulated by the transcription factor SERF1, is responsible for salt tolerance and is dependent on ROS [292].

To maintain ion homeostasis during salinity stress, plants employ different mechanisms. One of the mechanisms for tolerating salinity stress involves the transport of Na^+^ and Cl^−^ in the roots to prevent their excessive accumulation in the leaves. This process includes removing Na^+^ from the xylem and releasing ions back into the soil. If Na^+^ exclusion fails, it can have toxic effects on older leaves, leading to their premature death [293]. The concentration of Na^+^ in the rice leaves is linked with the salinity stress tolerance level in both *japonica* and *indica* rice varieties [294]. Maintaining a low cytosolic Na^+^/K^+^ ratio is important for maintaining ionic homeostasis and improving photosynthesis and overall plant growth [295,296]. During salinity stress, the accumulation of Na^+^ in the leaves and shoots of salt-tolerant varieties of rice is lower compared to salt-sensitive varieties [297,298]. It was also reported that the salt-tolerant cultivar Pokkali can reduce Na+ uptake into the cytosol and maintain lower cytosolic Na^+^ content by temporarily taking up Na^+^ into the cytoplasm and quickly extruding it into vacuoles. However, the salt-sensitive rice variety BRRI Dhan29 was unable to perform this function [299].

Due to osmotic stress, most organisms, including bacteria and plants, accumulate specific organic solutes, especially proline and sugars which are referred to as osmoprotectants [300,301]. Trehalose, a non-reducing sugar, stands out for having a unique property that protects biological molecules from dehydration stress. According to Garg et al., the production and accumulation of trehalose in transgenic rice can give the grain some resistance to the negative impacts of salinity and drought [302]. Glycine betaine, a potent solute containing quaternary ammonium, is found in several organisms. Though rice plants generally do not store glycine betaine, it has been shown that they may absorb exogenously and store it in their leaves to aid in sustaining PSII quantum yield when subjected to salt stress [289,303]. If the plant’s several defense strategies against salinity stress fail, it will implement programmed cell death (PCD) as a last-ditch effort to survive [304]. According to Liu et al.’s [305] findings, rice roots under salt stress had a well-regulated progression of cell death. This raised the possibility that the dead cells prevented salt exclusion by blocking the inflow of extra Na^+^ ions into the interior of roots and shoots. Another possibility is that the plant sheds cells to avoid unregulated cell death and the release of toxins to safeguard and maintain the growth of other cells [306].

#### 2.5.2. Molecular Response to Salinity Stress

Various proteins are involved in activating the tolerance mechanism against salt stress. They play different roles in the accumulation of MDA, antioxidants and osmoprotectants, ROS and Na^+^ homeostasis, and electrolyte leakage [289]. Certain WRKY TFs restrict the expression of *DREB1B* and *OsNAC1*, contributing to salt susceptibility [307].

TFs influence salt tolerance positively or negatively. *OsCOIN*, *OsbZIP71*, *OsbZIP23*, *OsDREB2A*, and *OsMYB2* are some of the salt-responsive TFs that may cause a variety of alterations in rice, such as a buildup of osmoprotectants and antioxidants and an upsurge in the activity of the Na^+^ and K^+^ transporters [308]. In rice, overexpression of these salt-responsive TFs promotes a higher survival rate of seedlings, reduces oxidative damage, and improves osmotic regulation [309,310]. On the contrary, *OsWRKY13*, one of the negative regulatory TFs, prevents the expression of the salt-responsive genes *SNAC1* and *ERD1*, thereby delaying the rice plants’ growth and development [311]. The expression of genes including *SNAC1*, *NCED4*, *Rab16D*, and *DREB1B* was suppressed by the transcriptional repressor *OsWRKY45-2*, and as a consequence, overexpression of *OsWRKY45-2* drastically lowered the survivability of rice cultivars under salt stress [312]. Liu et al. revealed two newly discovered genes (*LOC Os02g49700*, *LOC Os03g28300*) and five known genes (*OsMYB6*, *OsGAMYB*, *OsHKT1;4*, *OsCTR3*, and *OsSUT1*) connected with grain production and its associated attributes in rice cultivars exposed to saline stress conditions [313].

According to Rahman et al., maintaining lower shoot Na^+^ buildup is a standard method for preserving salt tolerance in rice [78]. These methods include sodium exclusion, effective toxic salt sequestration into older leaves and roots, compartmentalization of Na^+^ into vacuoles, and extrusion from cells. According to Wang et al., *OsHKT1;1*, *OsHAK10*, and *OsHAK16* were shown to be elevated in the leaves of old rice under salt stress [314]. These genes are integral to Na^+^ transport from the roots to the shoot. *OsHKT1;5* and *OsSOS1*, which promote Na^+^ exclusion from xylem vessels of roots, thereby lowering accumulation in the shoot, were downregulated, resulting in large quantities of Na^+^ in older leaves rather than young ones. Rice’s class 1 HKT transporter eliminates extra Na^+^ from the xylem, shielding the photosynthesis-dependent leaf tissues from the harmful effects of Na^+^. By mediating K^+^ absorption and transfer to sustain a high K^+^/Na^+^ ratio under salt stress, the K^+^ transporter genes *OsHAK1* and *OsHAK5* are stimulated by salt stress in rice [315]. When there is a higher concentration of Na^+^ in the cytosol, it is transported into the vacuole to prevent it from reaching toxic levels for enzyme reactions. Na^+^/H^+^ antiporters control this process. An increase in salt content activates the Na^+^/H^+^ antiporter action [316]. Two proton pumps, vacuolar H^+^-ATPase, and vacuolar H^+^-translocating pyrophosphatase, control the interchange of Na^+^/H^+^ in the vacuole. Modifying the vacuolar transporter levels can enhance rice’s tolerance to salinity [317]. According to a study, elevated *CYP94C2b* expression and concurrent jasmonate inactivation in rice are associated with salt tolerance [318]. Table 5 summarizes the key genes associated with salt stress tolerance in rice.

Arbuscular mycorrhizal fungus (AMF) symbionts aid the host plant development and ameliorate stress caused by abiotic factors. Under salt stress, the upland pigmented rice cv. Leum Pua (LP) infected with *Glomus etunicatum* produced total soluble sugars and free proline, which worked as osmolytes to preserve the flag leaf’s photosynthetic capacities, chlorophyll pigments, Chla fluorescence, and stomatal function. Leum Pua rice infected with *Glomus etunicatum* maintained yield characteristics and showed high anthocyanin content in the pericarp [333].

### 2.6. Heavy Metal Stress

Heavy metal pollution is a major contributor to harmful effects on plants, ecosystems, soil, and water. It is a significant factor in reducing the quality and yield of crops. Rice grown in paddy soils contaminated with heavy metals like arsenic (As), cadmium (Cd), lead (Pb), and mercury (Hg) is a major source of heavy metal intake for humans in many countries. This gradual buildup of heavy metals in rice grains and their subsequent entry into the food chain poses a severe risk to agriculture and public health [334]. Heavy metals have the potency to modify reactions that aid in generating ROS, ˙OH, and H_2_O_2_ within living cells. Nevertheless, when highly reactive radicals come into contact with water, they produce ˙OH, which can harm essential biomolecules within cells such as carbohydrates, lipids, amino acids, and DNA [335,336,337]. Therefore, it is necessary to comprehend how heavy metals interact with rice crops at all levels, from the cellular to the entire plant, and to develop effective strategies to reduce these stress reactions [338,339].

#### 2.6.1. Morphological and Physiological Responses to Heavy Metals

i. Arsenic

Arsenic can exist in various oxidation states in soil, the most prevalent of which are arsenides (As^3−^), arsenites (As^3+^), and arsenates (As^5+^). Depending on the species, arsenic can harm rice, with inorganic species being far more toxic than organic ones. As^5+^ and As^3+^ are the most prevalent inorganic species found in the rice plant, whereas monomethylarsonic acid (MMA) and dimethylarsinic acid (DMA) are the most occurring organic species [340]. As^3+^ is thought to be more mobile and hazardous than As^5+^ among inorganic entities. It can react with methyl groups in any oxidation state to create organic arsenic species. However, compared to inorganic arsenic species, the presence of organic species in paddy soil is substantially lower. The reduced form (As^3+^) predominates in anaerobic soil types, such as submerged rice fields, whereas As^5+^ (oxidized counterpart) predominates in aerobic soil environments, such as highland rice fields [341]. An increase in arsenic absorption will have a detrimental impact on plant development. Poor and lower germination rates of seeds, impaired plant growth, lower photosynthetic rates, sterility-related yield loss, low biomass production, and a physiological condition known as straight head disease are just a few of the symptoms that are brought on by arsenic toxicity in rice plants [342]. Reduced floret/spikelet sterility, decreased grain production, and, in severe cases, the absence of panicles or heads are some signs of this disease. Arsenic toxicity damages the chloroplast and photosynthetic processes by deteriorating the membrane structure. Arsenic affects the metabolism of proteins, lipids, and carbohydrates. More crucially, arsenic can increase the production of ROS that is greater than what can be scavenged, damaging plants through oxidative stress. Exposure of rice seedlings to As^5+^ promotes the formation of H_2_O_2_, whereas As^3+^ was shown to induce the formation of O_2_^−^ and H_2_O_2_, thereby causing lipid peroxidation [343]. When seedling roots are grown in an As^5+^ solution, APX activity is increased, reducing H_2_O_2_ through the ascorbate-glutathione cycle [344]. Similarly, the enzymatic antioxidants CAT, SOD, guaiacol peroxidase, chloroplastic ascorbate peroxidase, GR, and monodehydroascorbate reductase concentrations were raised for scavenging ROS developed in the presence of As^3+^ conditions [345].

ii. Cadmium

Cd is a trace element that is not necessary for plants but is widespread in the environment. Different anthropogenic operations such as smelting, mining, usage of synthetic phosphate fertilizers, and disposal of urban wastes lead to a rise in the levels of Cd in the environment that pose serious health risks to humans [346]. Recently, it has been found that Cd pollution in paddy soil poses a danger to rice quality [347]. Rice plants absorb Cd from the soil, eventually building up in the grains after several transit steps. Rice plant absorbs Cd from the ground through its roots, moves it to the shoots via xylem flow, reroutes it at nodes, and remobilizes it from the leaves. According to Huijie et al. [348], citrate, tartaric acid, and histidine were found to participate in root-to-shoot Cd transfer in the xylem actively. *Indica* cultivars often accumulate more significant amounts of Cd in their shoots and grains than *japonica* cultivars. Stomatal conductance, transpiration rate, leaf water content, vital minerals, water-soluble proteins, and enzyme- and non-enzyme-based antioxidants are all decreased due to Cd toxicity [349,350]. Cd poisoning reduced rice yield and grain quality by inducing changes in yield components (such as panicle number, spikelets per panicle, and spikelet setting percent). Excessive Cd has a deleterious impact on photosynthesis as it affects the photosynthetic pigments and disrupts electron transport mechanisms, interfering with chloroplast structure and Chl-protein complexes. This disruption causes a disturbance in Chl biosynthesis enzymes, the Calvin cycle, and water balance [351]. Cd prevents the formation of chlorophyll by inhibiting the enzyme δ-aminolevulinic acid dehydratase, which is present in rice seedlings. An increase in Cd concentration in the medium led to a higher accumulation of Cd in the seeds and the thiobarbituric acid reactive substance amount. It also caused a drastic decrease in the germination rate, shoot elongation, biomass, and water content of the rice [352].

Despite not being a direct cause, Cd can cause exorbitant accumulations of ROS when its concentration surpasses the plant tolerance level. This can occur through several mechanisms, comprising the exhaustion of ROS-scavenging enzymatic and non-enzymatic components, metabolic abnormalities during respiration, displacement of redox-active Fe from proteins, photorespiration, and CO_2_ assimilation [351,353].

iii. Lead

Pb is a non-essential element that may disrupt plant metabolism if taken up by the plant. In addition to interfering with roots’ ability to absorb minerals from the soil solution, Pb^2+^ ions also passively penetrate the roots of rice plants by following water streams that are moving through the soil. Pb is carried into the root epidermal cells from the soil and loaded into the root xylem vessels before being distributed to other plant organs [354]. In rice cultivars, a high Pb concentration (1.2 mM) results in a considerable decrease in plant height, tiller count, panicle count, and spikelet count per panicle [355]. Lead poisoning negatively affects photosynthetic activity by altering chloroplast structure, slowing the production of carotenoid, plastoquinone, and chlorophyll, and breaking up the electron transport chain. Additionally, it causes a CO_2_ shortage, which causes the stomata to close and Calvin cycle’s enzymatic activity to decrease. According to a study by Khan et al. [356], Pb poisoning does not affect root development but drastically reduces shoot length and biomass of rice in nitrogen or phosphorus-deprived seedlings. ROS are overproduced, and antioxidant enzyme activity fluctuates due to Pb toxicity in plants.

iv. Mercury

One of the environment’s most hazardous elements is Hg. Hg is a strong phytotoxin to plant cells at high concentrations and can cause injury and physiological disturbances. Hg preferentially accumulates on the roots of several plant species. As a result, the most toxic effects are observed at the roots. Under Hg stress, rice roots bind to proteins of 15–25 kDa, which results in irreparable harm to root development. Under Hg stress, rice roots altered the expression levels of the associated proteins [357]. When rice is grown on Hg-contaminated land, a significant amount of Hg is enriched into the grain, which is terrible for the rice’s consumers [358]. There are three different types of mercury: methylmercury (MeHg), inorganic mercury (Hg^2+^), and elemental mercury (Hg^0^) [359]. Hg is most bio-accumulative in the form of methylmercury MeHg. MeHg is the most harmful type of Hg to human and animal health [358]. The generation of MeHg in the rhizosphere soil and its buildup in rice are greatly influenced by moderate soil Hg content (3 mg kg^−1^). MeHg production in rhizosphere soil increases significantly at the blooming or filling stage, but rice leaves’ antioxidant systems show little impact [273]. The bulk of an individual rice grain’s Hg^2+^ by mass is found in the hull and bran. Conversely, white rice contains a large proportion of the more dangerous form of MeHg. Proteins contain MeHg, which is primarily coupled to cysteine in bran. This MeHg-cysteine relationship acts as a mobile nutrient during seed ripening and is actively transferred to the endosperm [360]. ROS, MDA content, and lipoxygenase activity are all considerably enhanced with increasing Hg levels in rice roots, which disturbs numerous cellular processes and hinders growth and development in rice plants [359].

#### 2.6.2. Biochemical Responses to Heavy Metals

An increased quantity of heavy metals like As, Hg, Pb, and Cd triggers ROS generation, leading to oxidative stress. This stress damages the plasma membrane and disrupts rice plants’ metabolism and physiological response. To combat oxidative stress, rice plants develop various defense strategies, such as activating the antioxidant defense system, ion homeostasis, osmolyte accumulation, osmoregulation, and excess production of signaling molecules [361,362]. In addition, in response to stress caused by heavy metals and metalloids, rice plants produce phytochelatins (PC), which are thiol-rich peptides [363]. For instance, rice leaves containing As-PC complexes reduce the amount of As^3+^ that may be transferred to the grain [364]. Similarly, under Cd stress, rice roots and leaves showed increased SOD, POD, CAT, GPX, and APX activity. Under Cd toxicity, rice also has higher levels of non-protein thiols like PCs and GSH to scavenge harmful free radicals [353]. In another experiment, rice showed an increase in the activity of CAT and POD under Pb poisoning. There was also an increase in the accumulation of proline and the content of sucrose with the rise in Pb concentration [355]

Recently, glutamate (Glu) has been found to participate in a signaling role in responses developed by plants toward abiotic stress [365]. In a study, glutamate supplementation was found to dramatically improve Cd-induced oxidative stress in rice with decreased levels of MDA, H_2_O_2_, O_2_^−^, proline, γ-aminobutyric acid, arginine, and higher activities of CAT, POD, and glutathione S-transferase. Roots of Cd-treated plants showed decreased expression of Cd-induced metal transporter genes OsNramp1, OsNramp5, OsIRT1, OsIRT2, OsHMA2, and OsHMA3 when supplemented with Glu [366]. According to Ahsan et al., 21 proteins were demonstrated to be engaged in defense and detoxification, antioxidant, protein biosynthesis, and germination activities in rice under Cd toxicity [367]. Hg stress raises the free Phe and Trp content and upregulated numerous genes related to aromatic amino acids. Chen et al. found that applying Phe and Trp to rice roots exogenously increases their tolerance to Hg and significantly decreases the concentration of ROS that Hg induces [368]. Additionally, research has shown that the formation of iron plaque on the roots of rice may serve as a protective barrier, reducing the absorption of Cd and As into the roots of the rice plant [369,370].

#### 2.6.3. Molecular Responses to Heavy Metals

**Figure 3:**
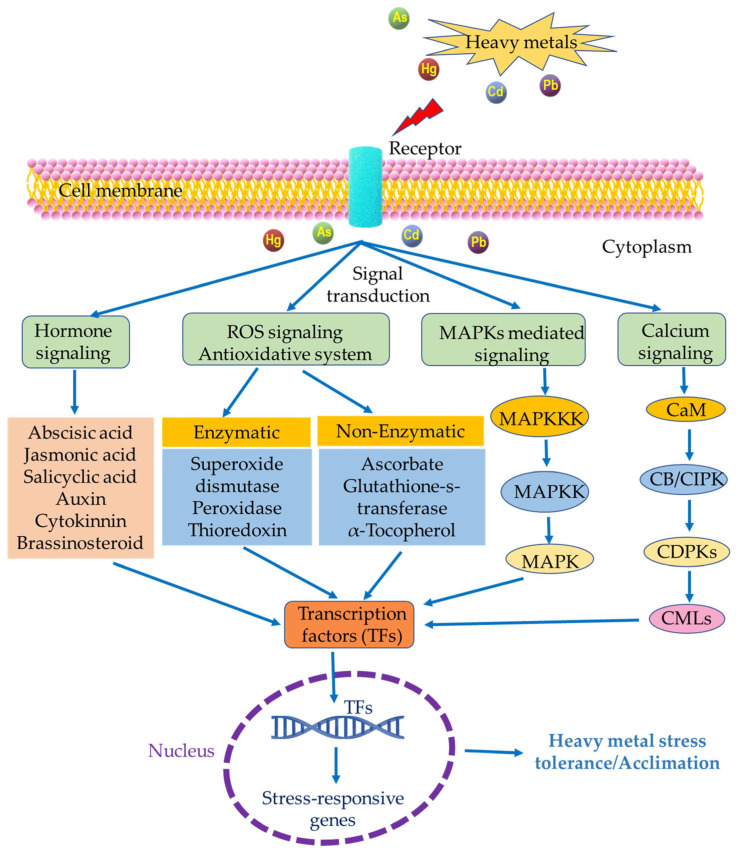
A schematic diagram showing a heavy metal stress signaling cascade that enhances stress-responsive gene expression in rice.

Heavy metal stress-related signal transduction is triggered by the recognition of stress signals by receptors/ion channels and then carried on by non-protein messengers such as calcium, hydrogen ions, and cyclic nucleotides (Figure 3).

The stress signals are relayed by several kinases and phosphatases, which in turn cause the expression of multiple TFs and the generation of metal-detoxifying peptides [371,372,373]. Heavy metals initiate various distinctive signaling pathways in plants, which include ROS signaling, calcium-dependent signaling, MAPK signaling, and hormone signaling that promote the expression of TFs and stress-responsive genes [344,372]. Calmodulins (CaM), calmodulin-like proteins, calcineurin B-like proteins, and CDPK are some of the calcium signaling sensors that monitor, process, and transmit changes in cytosolic Ca^2+^ content for the stress response. Individual sensors respond differently depending on the Ca^2+^ content [374,375]. Likewise, the MAPK signaling cascade also phosphorylates several TFs, including *NAC*, *MYC*, *MYB*, *bZIP, DREB*, and *ABRE*, which alters the expression of metal stress response genes [376,377]. For instance, Cd activates rice’s myelin basic protein (MBP) kinase and *OsMAPK2* genes [378]. Additionally, numerous research studies have displayed that the activation of MAPKs by heavy metals in rice is caused by ROS production, accumulation, and modification [372,379]. Furthermore, several phytohormone signaling pathways, especially ethylene, auxin, and JA, are affected by ROS. According to Singh and Shah, JA treatment enhanced rice’s ability to withstand Cd stress via improving antioxidant response [380]. When As^3+^ was applied to rice seedlings, comparative transcriptome analysis revealed modification in signal transduction, defensive responses, and hormonal signaling pathways, including ABA metabolism [381]. The results above strongly imply that changes in phytohormone levels alter how plants react to metal stress. Hence, it is crucial to comprehend the complex pathways through which metal stress is signaled in plants and the interconnections between them. This understanding is essential to unravelling the networks that plants employ to respond to stress. Numerous molecular research studies have examined how rice plants react to elevated levels of heavy metals. These research studies aim to enhance the ability of current rice cultivars to withstand heavy metal toxicity and offer valuable insights for incorporating these specific genes/traits into future breeding initiatives. Table 6 summarizes key genes associated with heavy metal tolerance in rice.

## 3. Conclusions

Abiotic stress is a significant factor restricting rice crop yield in many places of the world. Under the current climate change scenario, abiotic factors such as drought, heat, cold, submersion, salinity, and heavy metals are responsible for the sharp decline in rice yields. These abiotic stressors have a detrimental impact on various stages of plant growth and development, including germination, seedling establishment, lengths of root and shoot, plant height, blooming time, and ripening time. These stressors during both the vegetative and reproductive stages hinder the development of the plant’s panicles and the filling of grains, decreasing overall grain production and posing a risk to global food security. The combined application of genomics and QTL-based techniques has aided in identifying genes and loci that contribute to adaptation to abiotic stress in rice. These recently discovered molecular candidates have the potential to enhance rice physiological growth, reproductive development, and crop yields in challenging environments. However, in the future, research employing high-throughput phenotype determination and next-generation sequencing technology will help identify innovative potential genes responsible for regulating grain development under varied stress situations, paving the way for the breeding of climate-ready crops. In this review, we have discussed the developments in the current understanding of the defense mechanisms that rice employs to counteract various environmental stresses. Despite our vast knowledge in this area, there are still gaps in our understanding. Bridging these gaps will allow researchers to design plants that respond better to environmental stimuli such as drought, heat, cold, submersion, salinity, heavy metals, etc.

## Figures and Tables

**Table 1:** Identified genes linked to drought stress tolerance in rice.

Name of Genes	Function	Reference
*DRO1*	Stimulates the growth of roots, resulting in increased length and deeper penetration into the soil	[75]
*EcNAC67*	Enhances water content, postpones leaf curling, and increases the mass of roots and shoots	[78]
*DsM1*	Assists in removing reactive oxygen species and enhances drought resistance during the early growth (seedling) phase	[77]
*OsPYL/RCAR5*	Causes the closure of stomata and controls the weight of leaves	[33]
*OsDREB2B*	Length of roots and the amount of root growth	[73]
*OsNAC5*	Increases the size of the roots and improves the amount of grain produced	[79]
*SNAC1*	Enhances spikelet fertility	[80]
*OsLEA3-1*	Enhances grain yield	[81]
*OsbZIP23*	Increase grain yield	[82]
*OsbZIP72*	Enhancing tolerance to drought and increasing sensitivity to ABA (upregulating ABA)	[83]
*AP37*	Improves the process of seed filling and increases the weight of the grain	[84]
*OsNAC10*	Enhances resistance to drought during the vegetative phase, enhances root size, and enhances crop productivity	[79,85]
*EDT1/HDG11*	Increases water use efficiency, the buildup of compatible osmolytes, heightened antioxidant enzymatic activity, and improves photosynthesis	[86]
*AtDREB1A*	Osmolytes accumulation, maintenance of chlorophyll, increment in relative water content, and reduction in ion leakage	[87]
*OsCPK9*	Enhances drought tolerance in transgenics through improved stomatal closure and osmoregulation	[88]
*ADC*	Enhances resistance to drought by synthesis of polyamines such as putrescine and spermine	[61]
*OsOAT*	Enhances resistance to drought and promotes higher seed production	[89]
*OsTPS1*	Enhances rice seedling’s tolerance to drought, cold, and salinity stress	[90]
*P5CS*	Enhances biomass production under salinity and drought stresses	[91]
*HVA1*	Plasma membrane stability, increases leaf relative water content (RWC) and growth under drought stress	[92]
*Hrf1*	Drought resistance via antioxidants generation, ABA signaling, and regulating stomata closure	[93]
*JERF1*	Enhances drought resistance	[94]
*OsRDCP1*	Improves drought stress tolerance	[95]
*OsSDIR1*	Regulates stomata under drought stress	[96]
*OsSRO1c*	Regulates stomatal closure and enhances oxidative stress tolerance	[97]

**Table 2:** Identified genes linked to heat stress tolerance in rice.

Name of Genes	Function	Reference
*OsMYB55*	Enhances amino acids’ metabolic process, enhancing the ability to withstand high temperatures	[139]
*OsAREB1*	Controls abiotic stress-responsive gene expression utilizing an ABA-dependent mechanism	[143]
*OsHSF7*	Increases the expression of HSPs and other genes that protect against exposure to high temperatures, resulting in enhanced resistance to heat	[144]
*HSP101*	The effects of heat training in rice seedlings are prolonged by post-transcriptional interactions of *HSA32/HSP101* after heat treatment	[145]
*GAD3*	Participate in the ability to withstand high temperatures	[139]
*OsHTAS*	Improves rice’s ability to withstand heat by mediating stomata closure caused by H_2_O_2_	[146]
*TCM5*	Plays a vital role in the development of chloroplasts and the maintenance of PSII function in high temperatures	[147]
*EG1*	Enhances homeostasis in floral organs and the ability to withstand temperature changes by activating a pathway involving mitochondrial lipase in response to high temperatures	[147]
*OsTT1*	Breaks down poisonous denatured proteins while preserving the high-temperature response process	[127]
*TOGR1*	Plays a role in the normal processing of rRNA precursors at high temperatures and acts as a chaperone for the nucleolar SSU complex, crucial for cell growth in high-temperature environments	[148]
*OsHES1*	Plays a crucial part in adjusting to heat stress and ensuring the proper functioning of chloroplasts.	[149]
*OsAET1*	Plays a dual function in regulating the response to high temperatures through tRNA modification and control of translation	[150]
*OsNTL3*	Plays a crucial role in thermotolerance by interacting with *OsbZIP74*	[151]
*OsHsfA2c*	Involved in regulating the transcription of the HSP100 gene in the cytoplasm of rice	[152]
*OsHCI1*	Facilitates the nuclear export of target proteins, and its heterologous expression enhanced thermotolerance	[141]
*OsNSUN2*	Controls the mRNA modification of 5-methylcytosine (m5C), which improves mRNA translation efficiency and sustains normal development at higher temperatures	[153]
*OsTT3.1*	TT3.2 is ubiquitinated by TT3.1 for vacuolar degradation, and TT3.1 may function as a thermosensor	[154]
*OsTT3.2*	Chloroplasts rely on mature TT3.2 proteins to protect thylakoids against the detrimental effects of heat stress	[154]
*OsANN1*	Enhances SOD and CAT activity, controls H_2_O_2_ content and redox homeostasis, to provide cell protection against abiotic stress	[155]

**Table 3:** Identified genes linked to cold stress tolerance in rice.

Name of Genes	Function	Reference
*OsLTPL159*	Reduces the toxic effects of ROS, increases cell wall’s cellulose deposition, and increases osmolyte accumulation in rice, which increases the plant’s ability to withstand cold temperatures in its early seedling stages	[200]
*qPSST6*	Long-chain fatty acid production, involved in rice’s cold-tolerance during the booting stage	[201]
*OsCOIN*	Protein induced by cold enhances cold, drought, and salt tolerance	[202]
*Osa-MIR319a*	Increased leaf blade width	[203]
*OsGH3-2*	Regulates ABA and auxin levels during cold and drought stress	[204]
*OsMYB3R-2*	Regulates cell cycle (especially G2/M phase) to mediate cold tolerance in rice	[205]
*SNAC2*	Enhances cold and salt tolerance in rice	[206]
*OsDREB1F*	Enhances cold tolerance in rice	[207]
*ASR3*	Enhances cold/draught tolerance mediated by hormonal/sugar signaling	[208]
*OsFAD2*	An essential enzyme that raises grain yield and germination rate under LTS (low-temperature stress conditions)	[209]
*OsLti6b*	Produces hydrophobic protein in the ovaries and stamens of flowers undergoing cold treatment	[210]
*OsWRKY45*	Has a significant role in the signaling of ABA and serves as a means of communication between abiotic and biotic stresses	[211]
*OsRAN2*	GTPase that enhances cold tolerance through cell cycle regulation	[212]
*OsSPX1*	Participates in phosphate signaling as well as the interplay between the oxidative and cold stress tolerance mechanisms.	[213]
*OsDEG10*	Produces RNA-binding protein and has a key role in cold tolerance as well as response to other stresses (anoxia, photooxidative, and salinity)	[214]
*Oscrr6*	It has a key role in rice growth/photosynthesis at colder temperatures	[215]
*OsPIP2*	Participates in water homeostasis during cold stress tolerance	[216]
*OsPRP3*	Involved in the enhancement of cold tolerance in rice	[217]
*OsAsr1*	Involved in both vegetative and reproductive stages of cold tolerance	[218]
*MYBS3*	Modulates cold tolerance signaling pathways	[219]
*OVP1*	Involved in lowering malondialdehyde levels and increasing proline accumulation to increase tolerance to cold	[220]

**Table 4:** Identified genes linked to submergence stress tolerance in rice.

Name of Genes	Function	Reference
*OsACS1*	Involved in ethylene production and the rapid elongation of the stem in submerged rice	[257,258]
*OsACS5*	Involved in ethylene production and the rapid elongation of the stem in submerged rice	[257,258]
*SNORKEL1 (SK1)*	ERFs that modulate the internode elongation of deepwater rice during submergence	[247]
*SNORKEL2 (SK2)*	ERFs that regulate the internode elongation of deepwater rice during submergence	[247]
*Submergence 1A (SUB1A)*	Plant quiescence and plant survival under complete submergence	[249]
*SDI*	Involved in internode elongation	[254]
*OsHSD1*	Involved in underwater photosynthesis in submerged rice	[259]
*OsTPP7*	Involved in anaerobic germination	[260]
*AGPPase*	Promotes increased non-structural carbohydrate (NSC) buildup, which is accessible for a quick recovery after submersion	[261]
*EREBP1*	enhances resistance to submersion and facilitates better recovery from extended submersion	[262]
*CIPK15*	Involved in the regulation of sugar and energy production enabling growth of rice under floodwater	[263]

**Table 5:** Identified genes linked to salt stress tolerance in rice.

Name of Genes	Function	Reference
*OsCPK12*	Increases resistance to high salt levels by decreasing ROS buildup	[319]
*OsLOL5*	Enhance ROS scavenging and rice tolerance under salinity stress	[320]
*OsMAPK44*	Participates in ion homeostasis under salinity stress	[321]
*OsJRL40*	Increases antioxidant enzymatic activities and maintains the balance of Na^+^/K^+^ during salinity stress. Manages rice’s salt stress by regulating the expression of genes responsible for transporting Na^+^/K^+^, as well as genes involved in salt-responsive transcription factors and proteins	[322]
*OsSAPK4*	Modulates ion homeostasis as well as the growth and development of rice in a salinized environment	[323]
*OsKAT1*	Enhances rice’s salinity tolerance by enhancing K^+^ uptake and thus decreasing Na^+^ accumulation	[324]
*OsTPS8*	Controls the ability of rice to tolerate salinity stress by managing the levels of soluble sugars and regulating the activity of genes related to ABA signaling through the regulation of *SAPK9*	[325]
*OsBADH1*	Enhances salinity stress tolerance by positively regulating osmoprotectant biosynthesis	[326]
*OsMYB91*	Manages the growth of rice and its ability to tolerate salt stress.	[327]
*OsVP1 and OsNHX1*	Enhances the tolerance of salt by decreasing the accumulation of Na^+^ in leaves, photosynthesis activity, and increase root biomass	[328]
*OsHKT1;1, OsHKT1;4 and OsHKT1;5*	Enhance the tolerance of salt by decreasing the accumulation of Na^+^ in shoots when exposed to salt stress	[329,330,331]
*OsHAK5*	Enhance rice’s salinity tolerance by contributing to cation homeostasis	[332]

**Table 6:** Identified genes linked to heavy metals stress tolerance in rice.

Name of Genes	Function	Reference
*OsHAC1;1* and *OsHAC1;2*	Drastically influence limiting the accumulation of As in both the shoots and grains of rice	[382]
*OsNRAMP5*	Enhances resistance to the toxicity of Cd	[383]
*OsHMA3*	Enhances resistance to the toxicity of Cd	[384]
*OsABCG31*	Enhances resistance to the toxicity of Cd and Pb	[385]
*OsLCT1*	Enhances resistance to the toxicity of Cd Al	[386]
*OsSIZ*	Enhances resistance to the toxicity of Cd	[387]
*OsZIP1*	Enhances resistance to the toxicity of Cd, Zn,	[388]
*OsNAC5*	Enhances resistance to the toxicity of Cd and Pb	[79]
*OsMT1e*	Encodes a metal-detoxifying protein	[389]
*OsIRO2*	TF that modulates the activity of genes related to Fe balance in rice	[390]
*OsIRT1*	Participates in Cd absorption in rice. It is involved in Cd stress tolerance	[391]
*OsPCS1*	It is involved in detoxifying heavy metals and involved in Cd stress tolerance	[392]
*OsLCD*	Involved in Cd compartmentation	[393]
*OsSUV3*	Improved Cd and Zn stress tolerance	[394]
*OsSRK*	Increases the uptake and transfer of Cd	[395]
*OsHMA2*	Improves transfer of Cd from roots to shoots	[395]
*OsMYB45*	Improves Cd stress tolerance	[396]
*OsHB4*	Improves Cd accumulation and tolerance	[397]
